# 2-(4-Fluoro­phen­yl)-3-(4-pyrid­yl)pyrido[2,3-*b*]pyrazine

**DOI:** 10.1107/S1600536809037295

**Published:** 2009-09-26

**Authors:** Pierre Koch, Dieter Schollmeyer, Stefan Laufer

**Affiliations:** a Institute of Pharmacy, Department of Pharmaceutical and Medicinal Chemistry, Eberhard-Karls-University Tübingen, Auf der Morgenstelle 8, 72076 Tübingen, Germany; b Department of Organic Chemistry, Johannes Gutenberg-University Mainz, Duesbergweg 10-14, D-55099 Mainz, Germany

## Abstract

In the crystal structure of the title compound, C_18_H_11_FN_4_, the pyridopyrazine system makes dihedral angles of 45.51 (7) and 44.75 (7)° with the attached 4-fluoro­phenyl ring and the pyridine ring, respectively. The 4-fluoro­phenyl ring makes a dihedral angle of 54.54 (8)° with the pyridine ring. The pyridine ring part of the pyridopyrazine ring and the pyrazine ring of two *c*-glide-plane-related mol­ecules form π–π inter­actions. The angle between the planes is 2.09 (7)° and the distance between the centroids is 3.557 (1)Å.

## Related literature

For preparation of pyridopyrazines under microwave conditions, see: Zhao *et al*. (2004 ▶).
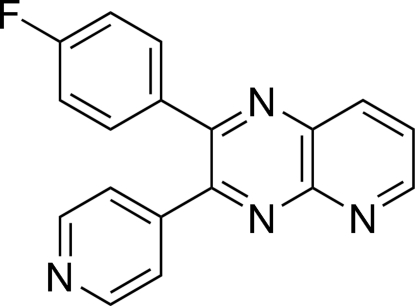


## Experimental

### 

#### Crystal data

C_18_H_11_FN_4_
*M* *_r_* = 302.31
Monoclinic, 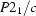

*a* = 17.222 (9) Å
*b* = 11.2199 (12) Å
*c* = 7.329 (4) Å
β = 91.80 (3)°
*V* = 1415.4 (10) Å^3^
*Z* = 4
Cu *K*α radiation
μ = 0.80 mm^−1^
*T* = 193 K
0.64 × 0.51 × 0.06 mm

#### Data collection

Enraf–Nonius CAD-4 diffractometer
Absorption correction: numerical (*CORINC*; Dräger & Gattow, 1971 ▶) *T*_min_ = 0.675, *T*_max_ = 0.949
2766 measured reflections
2677 independent reflections
2309 reflections with *I* > 2σ(*I*)
*R*_int_ = 0.029
3 standard reflections frequency: 60 min intensity decay: 2%

#### Refinement

*R*[*F* ^2^ > 2σ(*F* ^2^)] = 0.046
*wR*(*F* ^2^) = 0.134
*S* = 1.07
2677 reflections
209 parameters
H-atom parameters constrained
Δρ_max_ = 0.22 e Å^−3^
Δρ_min_ = −0.24 e Å^−3^

### 

Data collection: *CAD-4 Software* (Enraf–Nonius, 1989 ▶); cell refinement: *CAD-4 Software*; data reduction: *CORINC* (Dräger & Gattow, 1971 ▶); program(s) used to solve structure: *SIR97* (Altomare *et al*., 1999 ▶); program(s) used to refine structure: *SHELXL97* (Sheldrick, 2008 ▶); molecular graphics: *PLATON* (Spek, 2009 ▶); software used to prepare material for publication: *PLATON*.

## Supplementary Material

Crystal structure: contains datablocks I, global. DOI: 10.1107/S1600536809037295/nc2158sup1.cif

Structure factors: contains datablocks I. DOI: 10.1107/S1600536809037295/nc2158Isup2.hkl

Additional supplementary materials:  crystallographic information; 3D view; checkCIF report

## Figures and Tables

**Fig. 1.**
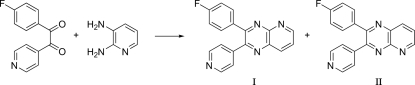
Synthesis of I and II.

**Fig. 2.**
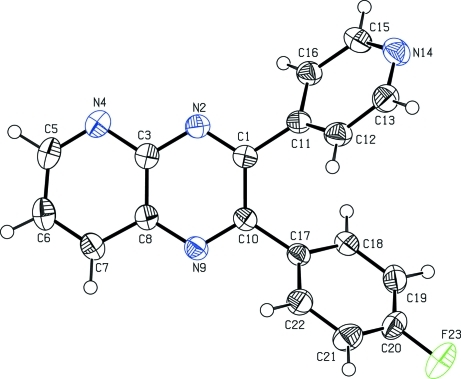
View of compound II. Displacement ellipsoids are drawn at the 50% probability level.

## supplementary crystallographic information

### Comment

The title compound, 2-(4-fluorophenyl)-3-(pyridin-4-yl)pyrido[3,2-*b*]pyrazine (II), was prepared in the course of our studies on pyridin-4-yl-substituted pyridopyrazines as potent p38 mitogen-activated protein (MAP) kinase inhibitors.

The microwave-assisted reaction of 1-(4-fluorophenyl)-2-(pyridin-4-yl)ethane-1,2-dione and 2,3-diaminopyridine yields two regioisomers, 3-(4-fluorophenyl)-2-(pyridin-4-yl)pyrido[2,3-*b*]pyrazine (I) and 2-(4-fluorophenyl)-3-(pyridin-4-yl)pyrido[3,2-*b*]pyrazine (II) (Figure I). The isomers were separated by flash-chromatography. To identify the two regioisomers *x*-ray analysis was used. In this article we present the *x*-ray data of the last eluted isomer II.

As might be expected the 4-fluorophenyl, the pyridine ring as well as the pyridopyrazine ring are planar (Figure 2). The pyridopyrazine ring makes dihedral angles of 45.51 (7)° and 44.75 (7)° to the 4-fluorophenyl ring and the pyridine ring, respectively. The 4-fluorophenyl ring makes a dihedral angle of 54.54 (8)° to the pyridine ring. The pyridine ring part of the pyridopyrazine ring and the pyrazine ring of two by c-glide plane related molecules forms π-π interactions. The angle between the planes is 2.09 (7)° and the distance of the centroids 3.557 (1) Å.

### Experimental

1-(4-Fluorophenyl)-2-(pyridin-4-yl)ethane-1,2-dione (113 mg, 0.5 mmol), and 2,3-diaminopyridine (54 mg, 0.5 mmol), and methanol/glacial acetic acid (2 ml, 9:1, V:V) were combined in a reaction vial. The reaction vessel was heated in a microwave reactor for 5 min at 433 K (initial power 250 W), after which a stream of compressed air cooled the reaction vessel to r.t. The solvent was removed under reduced pressure and the residue was purified by flash-chromatography (silica gel, petroleum ether/ethyl acetate 1–4 to 0–1) to yield 65 mg (43%) of II as a colorless solid. Suitable crystals of compound II for X-ray were obtained by slow evaporation at 298 K of a solution of n-hexane - diethyl ether (2–1).

### Refinement

Hydrogen atoms attached to carbons were placed at calculated positions with C—H = 0.95 Å (aromatic C-atom). All H atoms were refined in the riding-model approximation with isotropic displacement parameters (set at 1.2 times of the *U*_eq_ of the parent atom).

### Crystal data

**Table:**

C_18_H_11_FN_4_	*F*(000) = 624
*M**_r_* = 302.31	*D*_x_ = 1.419 Mg m^−^^3^
Monoclinic, *P*2_1_/*c*	Cu *K*α radiation, λ = 1.54178 Å
Hall symbol: -P 2ybc	Cell parameters from 25 reflections
*a* = 17.222 (9) Å	θ = 35–50°
*b* = 11.2199 (12) Å	µ = 0.80 mm^−^^1^
*c* = 7.329 (4) Å	*T* = 193 K
β = 91.80 (3)°	Plate, yellow
*V* = 1415.4 (10) Å^3^	0.64 × 0.51 × 0.06 mm
*Z* = 4	

### Data collection

**Table:**

Enraf–Nonius CAD-4 diffractometer	2309 reflections with *I* > 2σ(*I*)
Radiation source: rotating anode	*R*_int_ = 0.029
graphite	θ_max_ = 70.1°, θ_min_ = 2.6°
ω/2θ scans	*h* = 0→20
Absorption correction: numerical (CORINC; Dräger & Gattow, 1971)	*k* = 0→13
*T*_min_ = 0.675, *T*_max_ = 0.949	*l* = −8→8
2766 measured reflections	3 standard reflections every 60 min
2677 independent reflections	intensity decay: 2%

### Refinement

**Table:**

Refinement on *F*^2^	Secondary atom site location: difference Fourier map
Least-squares matrix: full	Hydrogen site location: inferred from neighbouring sites
*R*[*F*^2^ > 2σ(*F*^2^)] = 0.046	H-atom parameters constrained
*wR*(*F*^2^) = 0.134	*w* = 1/[σ^2^(*F*_o_^2^) + (0.0797*P*)^2^ + 0.3717*P*] where *P* = (*F*_o_^2^ + 2*F*_c_^2^)/3
*S* = 1.07	(Δ/σ)_max_ < 0.001
2677 reflections	Δρ_max_ = 0.22 e Å^−^^3^
209 parameters	Δρ_min_ = −0.24 e Å^−^^3^
0 restraints	Extinction correction: *SHELXL97* (Sheldrick, 2008), Fc^*^=kFc[1+0.001xFc^2^λ^3^/sin(2θ)]^-1/4^
Primary atom site location: structure-invariant direct methods	Extinction coefficient: 0.0012 (4)

### Special details

**Table:**

Geometry. All e.s.d.'s (except the e.s.d. in the dihedral angle between two l.s. planes) are estimated using the full covariance matrix. The cell e.s.d.'s are taken into account individually in the estimation of e.s.d.'s in distances, angles and torsion angles; correlations between e.s.d.'s in cell parameters are only used when they are defined by crystal symmetry. An approximate (isotropic) treatment of cell e.s.d.'s is used for estimating e.s.d.'s involving l.s. planes.
Refinement. Refinement of *F*^2^ against ALL reflections. The weighted *R*-factor *wR* and goodness of fit *S* are based on *F*^2^, conventional *R*-factors *R* are based on *F*, with *F* set to zero for negative *F*^2^. The threshold expression of *F*^2^ > σ(*F*^2^) is used only for calculating *R*-factors(gt) *etc*. and is not relevant to the choice of reflections for refinement. *R*-factors based on *F*^2^ are statistically about twice as large as those based on *F*, and *R*- factors based on ALL data will be even larger.

### Fractional atomic coordinates and isotropic or equivalent isotropic displacement parameters (Å^2^)

**Table:**

	*x*	*y*	*z*	*U*_iso_*/*U*_eq_	
C1	0.30818 (9)	0.56244 (13)	0.5692 (2)	0.0294 (4)	
N2	0.37936 (8)	0.58947 (12)	0.62301 (19)	0.0323 (3)	
C3	0.39843 (9)	0.70720 (14)	0.6323 (2)	0.0317 (4)	
N4	0.47243 (8)	0.73360 (13)	0.6904 (2)	0.0386 (4)	
C5	0.48914 (10)	0.84780 (17)	0.7036 (2)	0.0419 (4)	
H5	0.5405	0.8683	0.7432	0.050*	
C6	0.43761 (11)	0.94200 (16)	0.6641 (2)	0.0409 (4)	
H6	0.4539	1.0223	0.6795	0.049*	
C7	0.36428 (10)	0.91673 (14)	0.6039 (2)	0.0362 (4)	
H7	0.3283	0.9786	0.5750	0.043*	
C8	0.34280 (9)	0.79530 (14)	0.5851 (2)	0.0307 (4)	
N9	0.27022 (8)	0.76613 (11)	0.52296 (18)	0.0312 (3)	
C10	0.25245 (9)	0.65216 (13)	0.5120 (2)	0.0291 (3)	
C11	0.28858 (9)	0.43306 (14)	0.5734 (2)	0.0315 (4)	
C12	0.22105 (10)	0.39077 (15)	0.6474 (2)	0.0386 (4)	
H12	0.1837	0.4445	0.6930	0.046*	
C13	0.20869 (11)	0.26876 (16)	0.6541 (3)	0.0420 (4)	
H13	0.1619	0.2412	0.7048	0.050*	
N14	0.25842 (9)	0.18762 (13)	0.5938 (2)	0.0435 (4)	
C15	0.32369 (11)	0.22990 (15)	0.5235 (2)	0.0402 (4)	
H15	0.3602	0.1741	0.4801	0.048*	
C16	0.34126 (10)	0.34977 (14)	0.5101 (2)	0.0344 (4)	
H16	0.3885	0.3749	0.4585	0.041*	
C17	0.17368 (9)	0.62393 (13)	0.4370 (2)	0.0293 (3)	
C18	0.16111 (9)	0.53902 (13)	0.3005 (2)	0.0333 (4)	
H18	0.2036	0.4945	0.2571	0.040*	
C19	0.08724 (10)	0.51921 (15)	0.2280 (3)	0.0398 (4)	
H19	0.0786	0.4614	0.1348	0.048*	
C20	0.02664 (10)	0.58435 (16)	0.2928 (3)	0.0410 (4)	
C21	0.03648 (10)	0.66822 (17)	0.4283 (3)	0.0446 (4)	
H21	−0.0066	0.7112	0.4724	0.054*	
C22	0.11058 (10)	0.68841 (15)	0.4987 (2)	0.0379 (4)	
H22	0.1187	0.7472	0.5906	0.046*	
F23	−0.04531 (7)	0.56622 (13)	0.21986 (18)	0.0642 (4)	

### Atomic displacement parameters (Å^2^)

**Table:**

	*U*^11^	*U*^22^	*U*^33^	*U*^12^	*U*^13^	*U*^23^
C1	0.0316 (8)	0.0300 (8)	0.0268 (8)	0.0022 (6)	0.0028 (6)	0.0011 (6)
N2	0.0312 (7)	0.0329 (7)	0.0329 (7)	0.0013 (5)	0.0011 (5)	0.0018 (5)
C3	0.0319 (8)	0.0350 (8)	0.0283 (8)	−0.0016 (6)	0.0036 (6)	−0.0013 (6)
N4	0.0326 (7)	0.0447 (8)	0.0384 (8)	−0.0024 (6)	−0.0008 (6)	−0.0001 (6)
C5	0.0346 (9)	0.0514 (10)	0.0397 (10)	−0.0121 (7)	0.0005 (7)	−0.0046 (8)
C6	0.0436 (10)	0.0399 (9)	0.0396 (10)	−0.0126 (7)	0.0097 (8)	−0.0075 (7)
C7	0.0384 (9)	0.0304 (8)	0.0403 (10)	−0.0025 (6)	0.0081 (7)	−0.0029 (7)
C8	0.0312 (8)	0.0312 (8)	0.0299 (8)	−0.0019 (6)	0.0055 (6)	−0.0026 (6)
N9	0.0317 (7)	0.0280 (7)	0.0342 (7)	0.0007 (5)	0.0031 (5)	−0.0007 (5)
C10	0.0311 (8)	0.0279 (7)	0.0284 (8)	0.0009 (6)	0.0040 (6)	0.0000 (6)
C11	0.0338 (8)	0.0305 (8)	0.0298 (8)	0.0012 (6)	−0.0030 (6)	0.0046 (6)
C12	0.0367 (9)	0.0374 (9)	0.0418 (10)	0.0034 (7)	0.0020 (7)	0.0084 (7)
C13	0.0401 (9)	0.0421 (9)	0.0435 (10)	−0.0070 (7)	−0.0002 (7)	0.0115 (8)
N14	0.0523 (9)	0.0337 (7)	0.0443 (9)	−0.0024 (6)	−0.0008 (7)	0.0060 (6)
C15	0.0499 (10)	0.0308 (8)	0.0399 (10)	0.0044 (7)	0.0016 (8)	0.0018 (7)
C16	0.0366 (8)	0.0330 (8)	0.0334 (9)	0.0014 (6)	−0.0008 (6)	0.0029 (6)
C17	0.0312 (8)	0.0257 (7)	0.0311 (8)	0.0005 (6)	0.0016 (6)	0.0029 (6)
C18	0.0365 (8)	0.0282 (7)	0.0352 (9)	0.0005 (6)	0.0013 (7)	0.0009 (6)
C19	0.0453 (10)	0.0345 (8)	0.0394 (10)	−0.0070 (7)	−0.0033 (7)	−0.0014 (7)
C20	0.0295 (8)	0.0503 (10)	0.0431 (10)	−0.0074 (7)	−0.0027 (7)	0.0052 (8)
C21	0.0329 (9)	0.0533 (10)	0.0480 (11)	0.0059 (7)	0.0050 (8)	−0.0022 (8)
C22	0.0358 (9)	0.0388 (9)	0.0393 (10)	0.0027 (7)	0.0016 (7)	−0.0060 (7)
F23	0.0348 (6)	0.0915 (10)	0.0656 (8)	−0.0096 (6)	−0.0107 (5)	−0.0054 (7)

### Geometric parameters (Å, °)

**Table:**

C1—N2	1.311 (2)	C12—H12	0.9500
C1—C10	1.444 (2)	C13—N14	1.335 (2)
C1—C11	1.491 (2)	C13—H13	0.9500
N2—C3	1.362 (2)	N14—C15	1.338 (2)
C3—N4	1.363 (2)	C15—C16	1.383 (2)
C3—C8	1.412 (2)	C15—H15	0.9500
N4—C5	1.316 (2)	C16—H16	0.9500
C5—C6	1.404 (3)	C17—C22	1.393 (2)
C5—H5	0.9500	C17—C18	1.393 (2)
C6—C7	1.355 (3)	C18—C19	1.381 (2)
C6—H6	0.9500	C18—H18	0.9500
C7—C8	1.417 (2)	C19—C20	1.371 (3)
C7—H7	0.9500	C19—H19	0.9500
C8—N9	1.357 (2)	C20—F23	1.349 (2)
N9—C10	1.3167 (19)	C20—C21	1.375 (3)
C10—C17	1.482 (2)	C21—C22	1.380 (2)
C11—C12	1.383 (2)	C21—H21	0.9500
C11—C16	1.392 (2)	C22—H22	0.9500
C12—C13	1.386 (2)		
			
N2—C1—C10	122.18 (14)	C13—C12—H12	120.5
N2—C1—C11	115.41 (13)	N14—C13—C12	124.13 (17)
C10—C1—C11	122.41 (14)	N14—C13—H13	117.9
C1—N2—C3	117.45 (14)	C12—C13—H13	117.9
N2—C3—N4	116.60 (14)	C13—N14—C15	116.18 (15)
N2—C3—C8	120.38 (15)	N14—C15—C16	124.10 (17)
N4—C3—C8	123.01 (15)	N14—C15—H15	118.0
C5—N4—C3	115.75 (15)	C16—C15—H15	118.0
N4—C5—C6	125.61 (16)	C15—C16—C11	118.86 (16)
N4—C5—H5	117.2	C15—C16—H16	120.6
C6—C5—H5	117.2	C11—C16—H16	120.6
C7—C6—C5	119.09 (16)	C22—C17—C18	118.88 (15)
C7—C6—H6	120.5	C22—C17—C10	118.89 (14)
C5—C6—H6	120.5	C18—C17—C10	122.15 (14)
C6—C7—C8	118.08 (16)	C19—C18—C17	120.45 (16)
C6—C7—H7	121.0	C19—C18—H18	119.8
C8—C7—H7	121.0	C17—C18—H18	119.8
N9—C8—C3	121.61 (14)	C20—C19—C18	118.86 (16)
N9—C8—C7	119.96 (14)	C20—C19—H19	120.6
C3—C8—C7	118.43 (15)	C18—C19—H19	120.6
C10—N9—C8	117.69 (13)	F23—C20—C19	118.82 (17)
N9—C10—C1	120.54 (14)	F23—C20—C21	118.69 (17)
N9—C10—C17	116.01 (13)	C19—C20—C21	122.49 (16)
C1—C10—C17	123.44 (13)	C20—C21—C22	118.28 (17)
C12—C11—C16	117.74 (15)	C20—C21—H21	120.9
C12—C11—C1	122.42 (15)	C22—C21—H21	120.9
C16—C11—C1	119.75 (15)	C21—C22—C17	121.02 (16)
C11—C12—C13	118.99 (16)	C21—C22—H22	119.5
C11—C12—H12	120.5	C17—C22—H22	119.5
			
C10—C1—N2—C3	3.3 (2)	N2—C1—C11—C16	−43.4 (2)
C11—C1—N2—C3	−176.00 (14)	C10—C1—C11—C16	137.36 (16)
C1—N2—C3—N4	179.30 (14)	C16—C11—C12—C13	−0.4 (2)
C1—N2—C3—C8	0.0 (2)	C1—C11—C12—C13	−176.96 (16)
N2—C3—N4—C5	−178.11 (15)	C11—C12—C13—N14	0.3 (3)
C8—C3—N4—C5	1.2 (2)	C12—C13—N14—C15	0.0 (3)
C3—N4—C5—C6	0.3 (3)	C13—N14—C15—C16	−0.3 (3)
N4—C5—C6—C7	−1.3 (3)	N14—C15—C16—C11	0.2 (3)
C5—C6—C7—C8	0.6 (3)	C12—C11—C16—C15	0.2 (2)
N2—C3—C8—N9	−2.6 (2)	C1—C11—C16—C15	176.82 (15)
N4—C3—C8—N9	178.18 (15)	N9—C10—C17—C22	−45.2 (2)
N2—C3—C8—C7	177.49 (15)	C1—C10—C17—C22	135.38 (17)
N4—C3—C8—C7	−1.8 (2)	N9—C10—C17—C18	131.58 (16)
C6—C7—C8—N9	−179.16 (15)	C1—C10—C17—C18	−47.8 (2)
C6—C7—C8—C3	0.8 (2)	C22—C17—C18—C19	−0.1 (2)
C3—C8—N9—C10	1.6 (2)	C10—C17—C18—C19	−176.90 (15)
C7—C8—N9—C10	−178.43 (15)	C17—C18—C19—C20	−0.1 (2)
C8—N9—C10—C1	1.6 (2)	C18—C19—C20—F23	179.01 (16)
C8—N9—C10—C17	−177.79 (13)	C18—C19—C20—C21	−0.5 (3)
N2—C1—C10—N9	−4.3 (2)	F23—C20—C21—C22	−178.31 (17)
C11—C1—C10—N9	174.95 (15)	C19—C20—C21—C22	1.2 (3)
N2—C1—C10—C17	175.07 (14)	C20—C21—C22—C17	−1.3 (3)
C11—C1—C10—C17	−5.7 (2)	C18—C17—C22—C21	0.8 (3)
N2—C1—C11—C12	133.14 (17)	C10—C17—C22—C21	177.73 (16)
C10—C1—C11—C12	−46.1 (2)		
